# Associations Between Visual Accommodation and Cervical Muscle Activity and Symptomatology: A Systematic Review

**DOI:** 10.3390/jfmk10030252

**Published:** 2025-07-01

**Authors:** Miguel Ángel Lérida-Ponce, Miguel Ángel Lérida-Ortega, Ana Sedeño-Vidal, Alfonso Javier Ibáñez-Vera

**Affiliations:** 1 Department of Health Sciences, University of Jaen, 23071 Jaén, Spain; 2 Área de Gestión Sanitaria Norte de Jaén, Servicio Andaluz de Salud, 23700 Jaén, Spain

**Keywords:** visual accommodation, visual function, neck pain, cervical pain

## Abstract

**Objectives**: The aim of this study was to investigate the potential anatomical and physiological interconnections between the visual system and the cervical muscular system, as well as to examine the role of the visual system in the etiology and manifestation of cervical musculoskeletal pain or discomfort. **Methods**: A systematic review was conducted following the PRISMA guidelines, using databases such as PubMed, Scopus, Web of Science, CINAHL, and PEDro. The protocol was registered in PROSPERO. The included study designs comprised experimental studies, randomized controlled trials (RCTs), and pilot RCTs. **Results**: The literature search was conducted between January and May 2025 and yielded 51 studies across all databases. Seven experimental studies were finally included, all of which met the inclusion criteria and presented a mean methodological quality score of 5 on the PEDro methodological quality scale. The studies included data from a total of 308 participants (n = 261; 84.74% females). Subjects in the intervention group reported cervical pain or visual fatigue. **Conclusions**: Our results indicated a relationship between visual accommodation and increased electromyographic activity of the trapezius muscle, suggesting that accommodative stress may induce cervical muscle fatigue and pain.

## 1. Introduction

Alterations in the visual system and musculoskeletal disorders are significant public health issues that affect a large portion of the general population. Near visual tasks, reading, and screen use require precise coordination between the head-stabilizing muscles and the visual system. Head orientation in space and posture rely on the visual, vestibular, and proprioceptive systems [1].

During near visual work, the eyes are subjected to continuous accommodative and convergence effort, which leads to reduced use of distance vision and less frequent far-to-near-far focus shifts. This prolonged visual demand results in sustained activation of the extrinsic and intrinsic eye muscles, causing distortion and imbalance in visual behavior, which can lead to accommodative dysfunctions [2].

Accommodative anomalies and non-strabismic binocular dysfunctions are visual disorders that affect the binocular system and the patient’s visual performance. These visual disorders are associated with musculoskeletal discomfort in the cervical and shoulder regions, as well as headaches, often leading to the adoption of sustained non-ergonomic postures such as forward head position or asymmetric postures [3].

Uncorrected alterations in the visual system and the need for corrective lenses primarily increase the strain on the visual system and the head-stabilizing muscles, leading to visual fatigue, headaches, and cervicodorsal pain, as well as difficulty concentrating and poor coordination [1].

The visual effort based on the continued contraction of the ciliary muscles not only causes ocular discomfort but may also increase musculoskeletal tension, especially in the cervical and scapular regions, associated with increased activation of the trapezius muscle and the suboccipital musculature [4,5], thereby linking a visual disorder with a postural adaptation intended to maintain binocular function and visual comfort.

These discomforts may be physiologically connected or may result from postural adjustments unconsciously adopted as a response to visual fatigue or the use of inadequate optical correction. In this context, the cervical spine serves as a primary source for neuromuscular control [5]. Fatigue of the ocular muscles could lead to secondary changes in nerve activation, for example, linking a dysfunction in the sympathetic innervation of the eye in patients with whiplash injury to alterations in accommodation [6]. Therefore, this opens the possibility of a cross-dysfunction between the two systems.

The main objective of this systematic review is to determine the potential anatomical and physiological relationships between the ocular system and the cervical musculoskeletal system. The specific objectives are to justify the relationship between visual accommodation and the cervical muscular system, as well as its influence on musculoskeletal pain or discomfort related to the cervical region.

## 2. Materials and Methods

### 2.1. Clinical Literature Review

This systematic review and meta-analysis was conducted in accordance with the recommendations of the Preferred Reporting Items for Systematic Reviews and Meta-Analyses (PRISMA) [7] statement and the Cochrane Handbook for Systematic Reviews of Interventions [8]. The review was registered in PROSPERO (CRD420251053840).

### 2.2. Search Strategy

A literature search was carried out independently by two authors (MALP, ASV) from January to May 2025, across various health science databases, including PubMed (Medline), SCOPUS, Web of Science (WOS), CINAHL Complete, and the Physiotherapy Evidence Database (PEDro). To formulate the research question, the PICOS method proposed by the Cochrane Collaboration was used, as follows: P—population (patients with neck pain), I—intervention (visual tasks), C—comparison (pre-intervention and post-intervention results), and O—outcome (accommodation/vergence response, electromyographic muscle activity). For the search strategy, free-text terms such as “neck pain “ and “lens accommodation” were combined with MeSH terms and their synonyms indexed in PubMed MEDLINE. The search strategy incorporated MeSH terms and Boolean operators as follows: “((neck pain) OR (trapezius muscle activity)) AND ((ocular accommodation) OR (lens accommodation))”. No filters were applied for language or publication date. This phase was overseen by a third author with expertise in search strategies (AJIV). Table 1 shows the search strategy used.

### 2.3. Eligibility Criteria

Two authors (MALP, ASV) independently and carefully reviewed all records retrieved in the literature search by title and abstract. When a study was identified as potentially eligible in the review, the full text was read and examined by the two authors. Disagreements and uncertainties were resolved by a third author (AJIV).

The inclusion criteria were as follows: (1) experimental studies, randomized controlled trials (RCTs), or pilot RCTs; (2) patients with neck pan, upper limb pain, or visual dysfunctions based on the accommodative capacity of the lens; (3) studies that assessed the variability in visual accommodation and the perception of ocular and cervical fatigue and pain; (4) studies with a control group that received another type of intervention or no intervention; and (5) studies that evaluated outcome related to subjective symptoms of neck pain, visual accommodation, and cervical muscle activity.

The exclusion criteria were as follows: (1) a sample that was not entirely composed of patients with neck pain or studies where the visual accommodation was not alterations; (2) patients with concomitant conditions affecting cranio-cervical function and postural alterations; and (3) subjects who had ocular motility defects, strabismus, nystagmus, amblyopia, or any ocular or systemic disease that could affect the results.

### 2.4. Data Extraction

Data from the selected studies were extracted and coded into a data sheet standardized Microsoft^®^ Excel file, created for this review by two authors (M.A.L.P., A.S.V.). Any discrepancies were resolved by consulting a third author (A.J.I.V.).

After joint data comparison and review, a consensus on result interpretation was sought, ensuring validity and objectivity. From each study, the following data were extracted: (1) general features (authorship, publication date, country and funding); (2) patient characteristics (total sample size, number of participants in each group, age, and sex); (3) characteristics of the experimental and control groups (type of intervention, number of sessions); and (4) data related to variables (evaluated variable, test used, and time point of assessment).

### 2.5. Outcomes

The variables assessed in this systematic review were the following: the accommodative/vergence response (measured using an infrared refractometer); heart rate variability (measured through electrocardiography and electromyography); trapezius muscle activity (measured using electromyography); and ocular and cervical fatigue (evaluated using the Borg CR-10 Scale).

### 2.6. Risk of Bias and Quality of Evidence Assessments

The evaluation of the risk of bias in each included study and the quality of the evidence of the main findings was carried out by 2 authors independently (M.A.L.P. and A.S.V.). The methodological quality and the risk of bias of the included studies were assessed using the PEDro scale [9]. It consists of 11 items that assess the internal validity and the interpretability of trial results. The scale evaluates essential methodological aspects including randomization, blinding, and follow-up, with a score range from 0 to 10 (since one item is not scored). The total score indicates excellent (10–9 points), good (8–6 points), moderate (5–4 points), or poor (<3 points) methodological quality. Any discrepancies between researchers were resolved by a third investigator (A.J.I.V.).

## 3. Results

### 3.1. Study Selection

**Figure 1:**
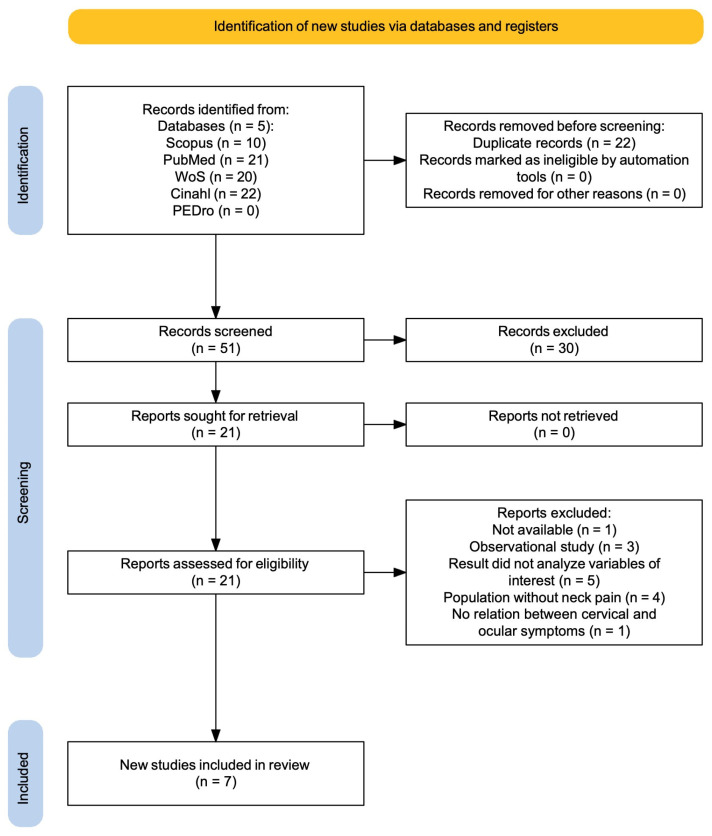
PRISMA flow diagram.

After removing duplicates, twenty-two references were excluded. Thirty studies were excluded based on the title/abstract, and fourteen studies were excluded based on the selection criteria. A total of twenty-one distinct articles were obtained, which were assessed for eligibility based on the inclusion criteria: three were excluded due to study design, five for not analyzing the variables of interest, four for involving healthy populations, and one for not linking cervical pain with ocular dysfunctions. Additionally, one potential article was not included as it did not have a digital version (published in 1985 and in Norwegian) [10]. A total of seven studies [11,12,13,14,15,16,17] met the eligibility criteria and were included in this systematic review. The PRISMA flow chart (Figure 1) shows the study selection process.

### 3.2. Characteristics of the Studies Included in the Review

The studies included [11,12,13,14,15,16,17] were conducted between 2010 and 2017. A total of 308 participants were provided by the included studies (150 in the control group; 158 in the experimental group) with a mean age of 18 ± 47 years (75.64% female). All the studies reported data on the immediate effects of demanding visual tasks, which varied in duration, distance, and visual condition. The sessions lasted between 10 and 30 min. Regarding the variables, the accommodative/vergence response was assessed using infrared technology, neck/shoulder discomfort and muscle fatigue were assessed using Borg’s CR-10 scale [17], muscle activity was measured with electromyography [11,12,13,14,15,16], and heart rate variability was measured using electrocardiography [12,15,16] to reduce disturbances from heart signals on raw electromyography and as an indicator of autonomic reactivity during the experiments. No external funding was reported in any of the studies. Table 2 shows the main characteristics of the included studies.

### 3.3. Assessment of Methodological Quality and Main Biases Identified

Table 3 reports the PEDro score for each study included in the review. The studies showed moderate methodological quality and a medium risk of bias (PEDro score of 5 points). None of the studies blinded the participants, evaluators, or therapists, nor was there any concealed allocation or masking, which increased the risk of performance biases.

### 3.4. Main Findings in Systematic Review

#### 3.4.1. Effects of Visual Work on the Accommodation Response

In six of the seven selected studies [11,12,14,15,16,17], a greater accommodative response was observed with negative lenses compared to neutral lenses. The study by Richter et al., 2015 [16] showed an insufficient response, as the accommodation of the lens did not need to compensate for the accommodative load under both binocular and monocular negative conditions. The study by Richter et al., 2010 [14], used Prism-Out lenses (which induce an external deviation of the eyes), showing an appropriate visual accommodation response. Only in the study by Richter et al., 2011 [15] was an inconsistency observed between accommodation and convergence with positive lenses. No significant differences were found between the symptomatic group and the control group in the selected studies, except for the study by Zetterberg et al., 2015 [11], where the accommodative response was greater in the control group (*p* = 0.049) compared to the group with cervical symptoms. Table 4 presents the primary outcomes with statistical data from the selected studies.

#### 3.4.2. Effects of Visual Work on Cervical Muscle Tone

Six of the seven selected studies [11,12,13,14,15,16] analyzed the effects of visual work on trapezius muscle activity. Three of the studies [13,14,15] showed that accommodative stress induced by the use of lenses with positive or negative load increased trapezius muscle tone (*p* = 0.001). Two studies [12,16] identified a significant correlation between accommodative stress and an increase in trapezius muscle tone, but only under binocular vision conditions (*p* = 0.009) according to Zetterberg et al., 2013 [12] and (*p* = 0.007) Richter et al., 2015 [16]. Under other visual conditions, trapezius activity remained low with no statistically significant differences, while one of the included studies found no significant differences in muscle tone across the different visual tasks (*p* = 0.1) [11]. In the six studies [11,12,13,14,15,16], muscle tone was quantified by recording electromyographic activity (Table 4).

#### 3.4.3. Effects of Visual Work on Heart Rate

In three of the selected studies [11,13,14] electrocardiography was not a primary variable but a technical tool to optimize the electromyographic recording. Conversely, in another three studies [12,15,16], electrocardiography, in addition to being used to optimize the electromyographic recording, was used to measure heart rate variability, thereby assessing the influence of the autonomic nervous system. Across these latter studies, the autonomic nervous system was not altered at any point during the experiment with any of the proposed visual tasks. Only in the study by Richter et al., 2015 [16] was a statistically significant decrease observed in the binocular vision condition with negative lenses (*p* = 0.024).

#### 3.4.4. Effects of Visual Work on the Perception of Fatigue and Pain

Only one of the included studies [17] analyzed the perception of fatigue and pain in the eyes, cervical region, and glenohumeral area. This study observed that, after accommodative stress induced by negative lenses, there was a significant increase in fatigue and pain in the eyes, cervical area, and glenohumeral region (*p* = 0.001). This variable also increased with positive lenses, although in this case, the differences were not significant (*p* = 0.3). In this study [17], the Borg CR-10 Scale, a numerical scale with verbal expressions used to quantify pain intensity, was employed to assess the perception of fatigue [18].

## 4. Discussion

All studies [11,12,13,14,15,16,17] have demonstrated a relationship between the visual system and the cervical musculoskeletal system. On the one hand, the authors have established a relationship between ocular accommodation and the cervical region [11,12,13,14,15,16]. These studies induced visual accommodation changes through the use of positive, negative, and neutral lenses. However, most of the reviewed literature did not directly ascertain whether ocular accommodation affects cervical conditions. The various authors described variations in trapezius electromyographic activity induced by changes in lens accommodation. However, the findings were inconsistent across studies. These findings suggest the possibility of increased cervical muscle tone as a mechanism to stabilize gaze in response to a mismatch in visual accommodation.

Previous studies observed a relationship between binocular vision and the neck system. Sánchez-González et al. [3] evaluated binocular vision status and confirmed a relationship between nonstrabismic binocular dysfunctions and musculoskeletal neck disorders. Giffard et al. [19] demonstrated a relationship between convergence insufficiency and cervical pain, demonstrating reduced ocular convergence in patients with cervical pain compared to asymptomatic subjects. Matheron et al. [20] reported head rotation in an attempt to compensate for the vertical deviation produced by a prism placed in front of the eye.

Other studies, such as that by Domkin et al. (2019) [21], observed that visually demanding tasks lead to increased trapezius muscle activity. Moreover, Richter et al. (2012) [22] emphasized that the patient’s accommodative effort is more closely related to trapezius EMG activity than the actual inability to focus.

Several authors have reported accommodative and vergence disorders in whiplash patients. Roca et al. [23] and Burke et al. [24] found that convergence and accommodation are reduced in whiplash patients. Ischebeck et al. [25] suggested that these patients exhibit altered eye reflexes and smooth pursuit movements, which may impair the coordination of the head and eyes. Stiebel-Kalish et al. [26] stated that whiplash patients exhibited convergence insufficiency and accommodative disorder symptoms. Ischebeck et al. [27] noted that severely impaired chronic neck pain patients have an elevated cervico-ocular reflex (COR) compared to healthy controls. These results are similar to those obtained in our study, supporting the hypothesis that there is an adaptive postural modification of the cervical spine related to vision.

Physiological processes that connect accommodative stress with activation of the trapezius muscle, such as the coordination between sensory and motor systems and mechanisms for postural control, can lead to postural changes including tilting or turning the head, lifting the chin, lowering it, or a mix of these, depending on the underlying cause [28]. According to Nucci et al., tension in the neck muscles may occur as a secondary effect when the head tilts to compensate for a vertical misalignment caused by dysfunction in the superior oblique muscle [29]. This head position can reduce the strain on the affected ocular muscles through reflexive responses. Although such an adjustment may support better visual function, over time, it may also result in musculoskeletal issues in the cervical region. From this perspective, discomfort or pain in the neck may arise as a consequence of the body’s effort to optimize visual performance. In this context, cervical disorders may develop due to sustained postural compensation aimed at maintaining visual comfort, highlighting the clinical implications of integrating optometric and postural interventions [30].

On the other hand, one of the selected studies [17] focused on cervical musculoskeletal symptoms and ocular symptoms. The Borg CR-10 Scale was measured before and after performing the visually demanding task, and it was concluded that a change in accommodative load led to an increase in ocular and cervical discomfort. This suggests that cervical pain may be caused by accommodative stress. In this regard, we found studies such as the one published by Lodin et al. (2012) [4], which concluded that a demanding visual exercise can lead to the onset of cervical and ocular symptoms. The study published by Sánchez-González et al. (2018) [2] determined a significant association between accommodative dysfunctions and the presence of pain in the cervical region, measured using the Neck Disability Index, the Visual Analogue Scale, cervical range of motion, deep flexor muscle activation score, and performance index.

While the findings of this work offer clinical relevance, further clarification is needed regarding the percentage of change in EMG trapezius activity during vision tasks. Future studies should address the median and standard deviation values of each group over time and the group interaction. It is also important to consider some limitations: the inclusion of a limited number of studies, the relatively small sample sizes in some of these studies, the local recruitment (all studies performed in Sweden), and the predominance of female participants may reduce the robustness and generalizability of our findings. Another limitation is the potential for publication bias to influence several of our results. Nonetheless, several studies seem to have used the same sample, and the quality of evidence reported was moderated. None of the studies presented random selection or blinding of participants, assessors, or therapists. Additionally, it is important to note that the possibility of patient follow-up could not be considered for the studies included due to limitations in their design. Future studies with larger sample sizes are necessary to elucidate the impact of the relationship between visual accommodation and trapezius tone.

## 5. Conclusions

Given the descriptive nature of the data and the lack of statistical synthesis, the findings of this systematic review suggest that there is an anatomical and physiological relationship between the ocular system and the cervical musculoskeletal system, indicating a possible influence on trapezius muscle tone due to visual demand. The limited differences between the control and symptomatic groups reflect that cervical pain does not worsen visual accommodation. However, insufficient accommodative capacity could potentially induce a painful muscular process in the cervical region. Further studies are required to enhance the generalizability, evidence base, and robustness of these findings.

## Figures and Tables

**Table 1:** Search Strategy.

Database	Search Strategy
**PubMed Medline**	((cervical pain) OR (cervicalgia) OR (neckache) OR (neck pain) OR (neck muscles) OR (trapezius muscle activity)) AND ((ocular accommodation) OR (lens accommodation))
**Cinahl**	AB (cervical pain or neck pain or cervicalgia or neckache or neck muscles or trapezius muscle activity) AND AB (ocular accommodation or lens accommodation)
**Web Of Science**	TOPIC (cervical pain or neck pain or cervicalgia or neckache or neck muscles or trapezius muscle activity) TOPIC (ocular accommodation or lens accommodation)
**PEDro**	Cervical pain and Ocular accommodation
**Scopus**	TITLE-ABS-KEY “cervical pain” OR “cervicalgia” OR “neckache” OR “neck pain” OR “neck muscles” OR “trapezius muscle activity” AND “ocular accommodation” OR “lens accommodation”

**Table 2:** The main characteristics of the included studies in the review.

Study	N (F/M)	D	Experimental Group			Control Group			Outcomes/Measured
			Sample	Intervention		Sample	Intervention		
			Ne (Age)	Tt	Ses	Ne (Age)	Tt	Ses	
Richter et al., 2010 (Sweden) [14] FN: Yes	28 (18/10)	Ne: chronic neck pain and/or professional oculomotor problems (asthenopia) Nc: healthy symptom	13 (32 ± 7)	Four near-to-far vision tasks under different visual conditions	1 ses of 5 min	15 (27 ± 8)	Four near-to-far vision tasks	1 ses of 5 min	Accommodative/vergence response (refractor) Heart rate variability (electrocardiography; electromyography) Trapezius muscle activity (electromyography)
Richter et al., 2011 (Sweden) [15] FN: Yes	28 (18/10)	Ne: chronic neck pain and/or professional oculomotor problems (asthenopia) Nc: healthy symptom	13 (32 ± 7)	Two near-to-far vision tasks under different visual conditions	1 ses of 5 min	15 (27 ± 8)	Two near-to-far vision tasks	1 ses of 5 min	Accommodative/vergence response (refractor) Heart rate variability (electrocardiography; electromyography) Trapezius muscle activity (electromyography)
Forsman et al., 2012 (Sweden) [13] FN: No	28 (18/10)	Ne: chronic neck pain and/or professional oculomotor problems (asthenopia) Nc: healthy symptom	13 (32 ± 7)	Near-to-far vision tasks under different visual conditions	1 ses Near 15 times and at Far 15 times	15 (27 ± 8)	Near-to-far vision tasks under different visual conditions	1 ses Near 15 times and at Far 15 times	Accommodative/vergence response (refractor) Heart rate variability (electrocardiography; electromyography) Trapezius muscle activity (electromyography)
Zetterberg et al., 2013 (Sweden) [12] FN: Yes	66 (54/12)	Ne: neck/shoulder pain in the last 12 weeks Nc: healthy symptom	33 (median age 39 [range 20–47])	Visually demanding near work at a computer screen under different visual conditions	1 ses 4 series of 7 min	33 (median age 39 [range 20–47])	Visually demanding near work at a computer screen under different visual conditions	1 ses 4 series of 7 min	Accommodative/vergence response (refractor) Heart rate variability (electrocardiography; electromyography) Trapezius muscle activity (electromyography)
Richter et al., 2015 (Sweden) [16] FN: Yes	66 (54/12)	Ne: chronic neck pain Nc: healthy symptom	33 (median age 39 [range 20–47])	Four near-to-far vision tasks under different visual conditions	1 ses 4 series of 7 min	33 (median age 39 [range 20–47])	Four near-to-far vision tasks under different visual conditions	1 ses 4 series of 7 min	Accommodative/vergence response (refractor) Heart rate variability (electrocardiography; electromyography) Trapezius muscle activity (electromyography)
Zetterberg et al., 2015 (Sweden) [11] FN: Yes	26 (17/9)	Ne: chronic neck pain and/or professional oculomotor problems (asthenopia) Nc: healthy symptom	12 (26 ± 8) 26 ± 8	Near-to-far vision tasks under different visual conditions	1 ses of 2.5 min	14 (32 ± 7)	Near-to-far vision tasks under different visual conditions	1 ses of 2.5 min	Accommodative/vergence response (refractor) Heart rate variability (electrocardiography; electromyography) Trapezius muscle activity (electromyography)
Zetterberg et al., 2017 (Sweden) [17] FN: Yes	66 (54/12)	Ne: chronic neck pain Nc: healthy symptom	33 (median age 39 [range 20–47])	Four near-to-far vision tasks under different visual conditions	1 ses of 7 min	33 (median age 39 [range 20–47])	Four near-to-far vision tasks under different visual conditions	1 ses of 7 min	Accommodative/vergence response (refractor) Heart rate variability (electrocardiography; electromyography) Ocular and cervical fatigue (Borg CR-10 scale)

FN: funding N: total sample size; D: diagnostic; F/M: female/male; Ne: experimental group sample size; Tt: Treatment; Ses: session; Nc: control group sample size; min: minutes.

**Table 3:** PEDro methodological quality scale.

Author	C1	C2	C3	C4	C5	C6	C7	C8	C9	C10	C11	Total Score
Richter et al., 2010 [14]	✓	✓	✗	✓	✗	✗	✗	✓	✗	✓	✓	5
Richter et al., 2011 [15]	✓	✓	✗	✓	✗	✗	✗	✓	✗	✓	✓	5
Forsman et al., 2012 [13]	✓	✓	✗	✓	✗	✗	✗	✓	✗	✓	✓	5
Zetterberg et al., 2013 [12]	✓	✓	✗	✓	✗	✗	✗	✓	✗	✓	✓	5
Zetterberg et al., 2015 [11]	✓	✓	✗	✓	✗	✗	✗	✓	✗	✓	✓	5
Richter et al., 2015 [16]	✓	✓	✗	✓	✗	✗	✗	✓	✗	✓	✓	5
Zetterberg et al., 2017 [17]	✓	✓	✗	✗	✗	✗	✗	✓	✓	✓	✓	5

C: criteria; ✓: yes; ✗: no.

**Table 4:** Summary of primary outcomes from the selected studies.

Reference	Variable/Condition	Main Results (Experimental Group)
Richter et al., 2010 [14]	Accommodative error (D)/EMG (% RVE)	Binocular with −3.5 D: r^2^ =0.38; *p* = 0.0012 Binocular with 0.0 D: r^2^ = 0.003; *p* = 0.79 Binocular with 1–2 Prism D: r^2^ = 0.07; *p* = 0.203 Binocular with +3.5 D: r^2^ = 0.316; *p* = 0.0022
Richter et al., 2011 [15]	Response diopters (D)/EMG (% RVE)	Binocular with −3.5 D: r^2^ = 0.25; *p* = 0.013
		Binocular with 0.0 D: r^2^ = 0.016; *p* = 0.563
	Accommodative error (D)/EMG (% RVE)	Binocular with −3.5 D: r^2^ = 0.3686 *p* = 0.001
		Binocular with 0.0 D: r^2^ = 0.0018; *p* = 0.845
Forsman et al., 2012 [13]	EMG/refraction signals	R(tau) = 0.019; *p* = 0.001
Zetterberg et al., 2013 [12]	Accommodation response (D)/EMG (%RVE)	Binocular with −3.5: r = 0.377; *p* = 0.017
		Monocular with −3.5: r = 0.147; *p* = 0.326
		Monocular neutral with 0.0 D: r = −0.018; *p* = 0.897
		Monocular with +3.5: r = 0.088; *p* = 0.524
Zetterberg et al., 2015 [11]	Trapezius muscle activity (in %RVE)	Neutral lenses: 1.82 [0.79, 2.86]; *p* = 0.034 Negative −3.5 D lenses: 2.36 [0.72, 3.99]; *p* > 0.1 Positive +3.5 D lenses: 1.74 [0.28, 3.21]; *p* = *p* > 0.1
Richter et al., 2015 [16]	Trapezius muscle activity (% RVE) during the visual tasks	Binocular −3.5 D: *p* = 0.007 Monocular −3.5 D: *p* = 0.048 Monocular 0 D: *p* = 0.043 Monocular +3.5 D: *p* > 0.5
Zetterberg et al., 2017 [17]	Accommodation response	Binocular −3.5 D: 3.39 (2.09) Monocular −3.5 D: 3.68 (2.00) Monocular 0 D: 1.51 (0.74) Monocular +3.5 D: 0.97 (0.99)
	BOR’s scale internal eye discomfort	Binocular −3.5 D: 3.0 (0 ± 9.0) * Monocular −3.5 D: 2.0 (0 ± 7.0) Monocular 0 D: 2.0 (0 ± 7.0) Monocular +3.5 D: 3.0 (0 ± 9.0)
	BOR’s scale external eye discomfort	Binocular −3.5 D: 3.0 (0 ± 7.0) Monocular −3.5 D: 2.5 (0.3 ± 7.0) Monocular 0 D: 2.0 (0 ± 7.0) Monocular +3.5 D: 2.5 (0 ± 7.0)
	BOR’s scale neck/shoulder discomfort	Binocular −3.5 D: 3.0 (1.0 ± 9.0) Monocular −3.5 D: 3.0 (0.5 ± 10.0) Monocular 0 D: 3.0 (1.0 ± 10.0) Monocular +3.5 D: 3.0 (0.5 ± 9.0)

D: diopters; EMG: electromyography; RVE: reference voluntary electrical activity; r-value: Pearson correlation coefficient; *: values presented are medians with the range in brackets.

## Data Availability

The data of this review are available upon request to the corresponding author.
